# Real-World Comparison of Overall Survival Among Patients With and Without Inherited Retinal Diseases

**DOI:** 10.3390/vision10010015

**Published:** 2026-03-01

**Authors:** Byron L. Lam, Carlos E. Mendoza-Santiesteban, Dominic Pilon, Dejan Milentijevic, Laura Morrison, Samuel Schwartzbein, Claire Vanden Eynde, Marie-Hélène Lafeuille, Patrick Lefebvre, Ninel Z. Gregori

**Affiliations:** 1 Mark J. Daily Inherited Retinal Disease Research Center, Bascom Palmer Eye Institute, Miami Miller School of Medicine, Miami, FL 33136, USA; 2 Analysis Group, Inc., Montréal, QC H3B 0G7, Canada; 3 Johnson & Johnson, Titusville, NJ 08560, USA

**Keywords:** blindness, electronic health records, inherited retinal diseases (IRDs), mental health conditions, mortality, physical comorbidities, overall survival

## Abstract

This study compared real-world overall survival and the risk of physical comorbidities and mental health conditions among patients aged <65 years with versus without inherited retinal diseases (IRDs) in the United States (US). Optum^®^ Electronic Health Record data (January 2014–January 2023) were evaluated for IRD (patients with ≥2 medical visits with an IRD diagnosis; index date: second such medical visit) and non-IRD (patients without an IRD diagnosis; index date: random medical visit) cohorts. Baseline demographics were balanced between cohorts using propensity score matching (2:1). Outcome measures were overall survival (date of death due to any cause) and presence of physical comorbidities and mental health conditions (medical visit with a corresponding diagnosis code). In total, 4594 patients with IRD were matched to 9188 patients without IRD (mean age: 38.7 vs. 38.2 years, 53.9% vs. 55.1% female, mean follow-up: 53.1 vs. 52.8 months). Over 84 months, patients with versus without IRD had a 24% higher risk of death (overall survival: 95.8% vs. 96.7%; hazard ratio: 1.24; 95% confidence interval: 1.00–1.53; *p* = 0.046) and were at significantly higher risk for each evaluated physical comorbidity and mental health condition (all *p* < 0.05). The development of novel therapies is thus needed to address the clinical burden of IRD.

## 1. Introduction

Inherited retinal diseases (IRDs) are a heterogeneous group of conditions associated with over 300 genes that cause retinal cell degeneration and can lead to progressive vision loss and blindness [1,2,3]. Among some of the most prevalent IRDs are retinitis pigmentosa, Stargardt disease, Leber congenital amaurosis, cone-rod dystrophy, Best disease, X-linked retinoschisis, and achromatopsia [4]. More than 2 million people worldwide are affected by IRDs, making them among the most common causes of blindness among the working-age population [5,6]. In patients with retinitis pigmentosa, the most common group of IRDs, more than three-quarters of individuals present with symptoms and are diagnosed by the age of 30 years [7].

So far, there is only one Food and Drug Administration (FDA)-approved treatment for an IRD. Voretigene neparvovec-rzyl (Luxturna) is indicated for patients with biallelic RPE65 mutation-associated retinal degeneration and has been shown to improve light perception and mobility in dim environments among this population [8,9]. For the vast majority of patients with IRD, however, there are no approved therapies to halt disease progression or restore vision [10,11]. Novel genetic treatments, stem-cell-based therapies, and retinal prostheses have been in clinical development for the past two decades, though most have yet to receive regulatory approval [10,11].

Until effective and beneficial therapies are available for patients with IRD, it is essential to understand the prevalence of physical comorbidities and mental health conditions that may impact patients’ lives and design mitigating clinical approaches. In the United States (US), patients experiencing vision loss are more likely to develop mental health conditions such as depression and anxiety and to be less physically active than those without a visual impairment [12,13,14,15]. Prior research has also found higher all-cause mortality among US patients with vision loss relative to those with unimpaired vision [16,17], though these studies were not specific to a population diagnosed with an IRD. There is some research suggesting that patients with IRD may have a lower overall survival and increased likelihood of physical comorbidities compared to those without IRD [18,19], though the evidence in the US is limited. To mitigate this knowledge gap, the current study aimed to compare overall survival, as well as the presence of common physical comorbidities and mental health conditions, among patients aged <65 years diagnosed with IRD, relative to those without IRD, using a large US-based electronical health record (EHR) database.

## 2. Materials and Methods

### 2.1. Data Source

Clinical and administrative data from 1 January 2014 to 15 January 2023 were obtained from the Optum^®^ (Eden Prairie, MN, USA) de-identified Electronic Health Record data set (Optum^®^ EHR), a longitudinal clinical repository derived from numerous large healthcare provider organizations across the US that encompasses over 106 million patients. Data elements in Optum^®^ EHR include patient demographics, medications prescribed and administered, immunizations, comprehensive lab results, microbiology results, vital signs, clinical and inpatient stay administrative data, and coded diagnoses and procedures. As this study involved only the secondary use of data that were de-identified in compliance with the Health Insurance Portability and Accountability Act (HIPAA), specifically, 45 CFR § 164.514, this study was considered exempt research under 45 CFR § 46.104(d)(4).

### 2.2. Study Design

**Figure 1:**
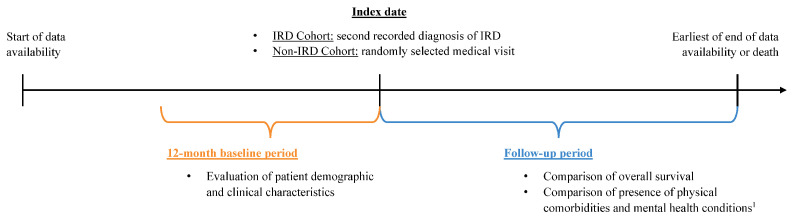
Study design. Abbreviation: IRD: inherited retinal disease. Note: ^1^. The following physical comorbidities and mental health conditions were included for comparison: chronic pulmonary disease, diabetes, peripheral vascular disease, congestive heart failure, stroke (including transient ischemic attack), cerebrovascular disease, myocardial infarction, anxiety disorders, depression, suicidal attempt or ideation, and trauma or stressor-related disorders.

The study employed a retrospective propensity score-matched cohort design. Adult patients were included in the IRD cohort if they had ≥2 medical visits with a diagnosis for IRD during the study intake period, which spanned from 1 January 2015, until 15 January 2022 (Figure 1). Patients were included in the non-IRD cohort if they had no diagnoses of IRD at any time and ≥1 medical visit during the study intake period.

Among the IRD cohort, the index date was defined as the date of the second medical visit with a diagnosis for IRD during the study intake period, to ensure IRD was accurately diagnosed and to minimize misclassification due to a rule-out diagnosis (see Table S1 for list of codes). For the non-IRD cohort, the index date was a randomly selected medical visit during the study intake period to identify a control cohort representative of individuals without IRD, rather than to match cohorts on prior healthcare utilization. The study intake period was chosen to allow 1 year of data availability before and after the index date to assess baseline characteristics and outcomes and to ensure study recency. The baseline period, defined as the 12 months prior to the index date, was used to evaluate patient demographics and clinical characteristics. Patients were followed from the index date to either the date of death or the end of data availability. This follow-up period was used to assess overall survival and the presence of physical comorbidities and mental health conditions.

**Figure 2:**
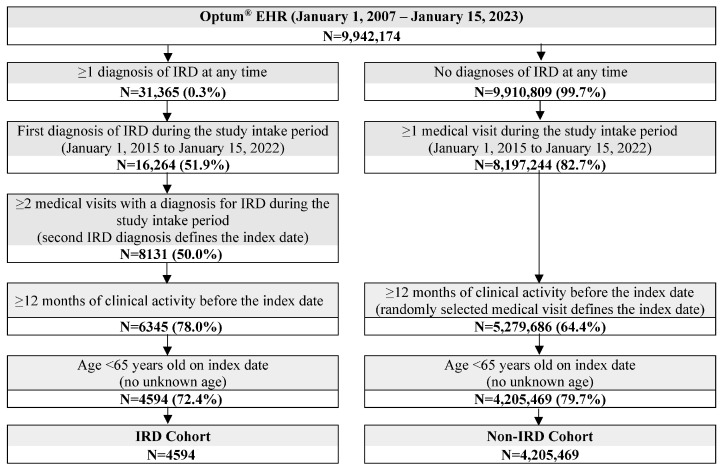
Sample selection. Abbreviations: EHR: electronic health record; IRD: inherited retinal disease.

Among both cohorts, patients were required to be <65 years of age on the index date and to have ≥12 months of clinical follow-up before the index date (Figure 2). Patients aged ≥65 years or with an unknown age were excluded to avoid the increased likelihood of having received diagnoses for IRD prior to the start of data availability, while patients with <12 months of clinical activity were excluded to gather a more complete baseline clinical profile of each patient.

### 2.3. Study Measures

Patient characteristics included demographic characteristics evaluated on the index date and clinical characteristics evaluated during the 12-month baseline period. Outcomes included overall survival and the presence of physical comorbidities and mental health conditions assessed during the follow-up period. Overall survival was defined as the time from the index date to the date of death from any cause. Patients who did not have a date of death were censored at the end of data availability.

The presence of selected physical comorbidities and mental health conditions followed a prevalence approach and was defined as a medical visit with a corresponding diagnosis code, regardless of the presence of the medical condition prior to the index date. Eleven physical comorbidities and mental health conditions were assessed, including anxiety disorders, cerebrovascular disease, chronic pulmonary disease, congestive heart failure, depression, diabetes, myocardial infarction, peripheral vascular disease, stroke including transient ischemic attack (TIA), suicidal attempt or ideation, and trauma-related or stressor-related disorders. These comorbidities and conditions were selected based on their comorbid nature with IRD, prior research evaluating the onset of these conditions among patients with IRD, as well as those with severe visual impairment, and clinical input from key opinion leaders [12,13,15,19,20]. The diagnosis codes used to identify each condition are provided in Table S2.

### 2.4. Statistical Analyses

Propensity score matching (PSM) was used to balance baseline demographic characteristics between the IRD and non-IRD cohorts. Each patient in the IRD cohort was matched with two patients in the non-IRD cohort based on probability estimates from a logistic regression model. Propensity scores were calculated based on the following variables: age, sex at birth, race, ethnicity, region, insurance type, and the year and month of the index date. The balance of baseline characteristics after matching was assessed using standardized differences, where <10% was considered well-balanced [21].

Baseline characteristics were reported for both unmatched and matched cohorts using means, standard deviations (SD), and medians for continuous variables; frequencies and proportions were used for categorical variables. Overall survival was reported using Kaplan–Meier (KM) rates for each 12-month interval during the follow-up period and was compared between matched cohorts using hazard ratios (HRs) from Cox-proportional hazards models, including the corresponding 95% confidence intervals (CIs) and *p*-values. Differences in the presence of physical comorbidities and mental health conditions during the follow-up period were compared between matched cohorts using odds ratios (ORs) and 95% CIs and *p*-values obtained from logistic regression models.

## 3. Results

### 3.1. Patient Characteristics

A total of 4594 patients with IRD were included in the study. Among a total of 4,205,469 patients meeting the study inclusion criteria for the non-IRD cohort, 9188 patients were matched to the IRD cohort.

Baseline demographic and clinical characteristics were well balanced between cohorts after matching. The mean age in the IRD cohort was 38.7 years, and 53.9% of patients were female, compared to 38.2 years and 55.1% female in the non-IRD cohort (Table 1). The majority of patients in both cohorts were Caucasian (IRD: 75.3%; non-IRD: 74.6%), not Hispanic (IRD: 83.8%; non-IRD: 83.3%), and from the Midwest region (IRD: 54.9%; non-IRD: 54.3%). Among the IRD cohort, the mean time between the first and second medical visits with a diagnosis of IRD was 7.4 months, with a median time between diagnoses of 2.0 months.

Among the matched cohorts, the mean Quan-Charlson Comorbidity Index (Quan-CCI) was 0.5 and 0.4 in the IRD and non-IRD cohorts, respectively (Table 2). During the 12-month baseline period, the most prevalent physical comorbidities among each cohort were chronic pulmonary disease (IRD: 10.9%; non-IRD: 8.9%) and diabetes (IRD: 9.4%; non-IRD: 7.1%). The most prevalent mental health conditions were anxiety disorders (IRD: 12.2%; non-IRD: 9.8%), followed by depression (IRD: 12.1%; non-IRD: 9.6%), and suicidal attempt or ideation (IRD: 3.4%; non-IRD: 2.5%).

### 3.2. Overall Survival

**Figure 3:**
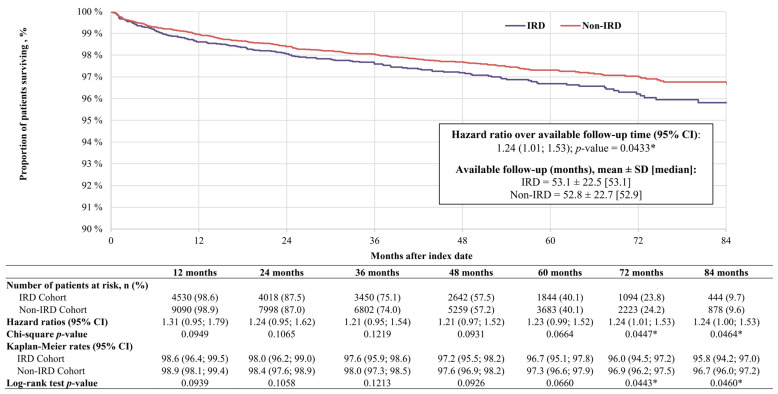
Kaplan–Meier rates of overall survival. Abbreviations: CI: confidence interval; IRD: inherited retinal disease; SD: standard deviation. * Indicates statistical significance, defined as *p* < 0.05.

Over an average follow-up period of ~53 months, patients with IRD had a 24% increased risk of death relative to patients without IRD (HR: 1.24, 95% CI: 1.01–1.53, *p* = 0.0433; Figure 3). Relative to the non-IRD cohort, the risk of death was numerically higher for patients with IRD at each 12-month interval and ranged from an increased risk of 21% to 31%. The increased risk among the IRD versus non-IRD cohort reached statistical significance at 72 months (HR: 1.24, 95% CI: 1.01–1.53, *p* = 0.0447) and 84 months (HR: 1.24, 95% CI: 1.00–1.53, *p* = 0.0464) after the index date, at which point the overall survival rate was 95.8% among the IRD cohort and 96.7% among the non-IRD cohort (*p* = 0.0460).

### 3.3. Presence of Physcial Comorbidities and Metnal Health Conditions

**Figure 4:**
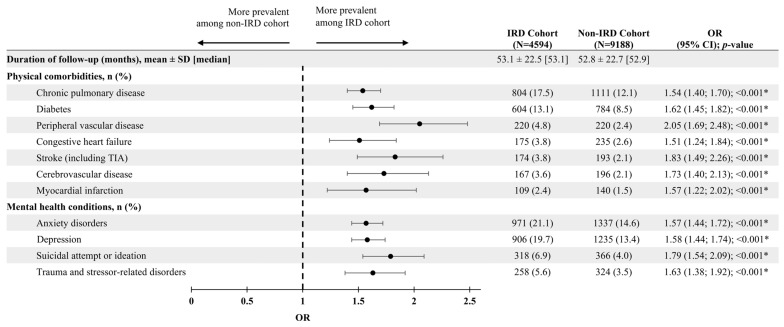
Presence of physical comorbidities and mental health conditions during the follow-up period. * Indicates statistical significance, defined as *p* < 0.05.

During the follow-up period, patients with IRD had a statistically significant higher likelihood of having at least one diagnosis or record for all 11 selected physical comorbidities and mental health conditions compared to patients without IRD (all *p* < 0.05; Figure 4). The increased likelihood of having each selected physical comorbidity or mental health condition in the IRD versus non-IRD cohort ranged from 51% (congestive heart failure) to 105% (peripheral vascular disease). The physical comorbidities and mental health conditions with the highest prevalence among both cohorts were anxiety disorders (IRD: 21.1%; non-IRD: 14.6%), depression (IRD: 19.7%; non-IRD: 13.4%), chronic pulmonary disease (IRD: 17.5%; non-IRD: 12.1%), and diabetes (IRD: 13.1%; non-IRD: 8.5%).

## 4. Discussion

This real-world study used integrated clinical and medical administrative EHR data from a large sample of patients with a diverse representation of geographic areas, providers, and payers in US clinical practice to compare the overall survival of patients aged <65 years with versus without IRD, finding that over an average follow-up of 53 months, the risk of death was significantly higher among patients with IRD relative to those without IRD. Further, patients with IRD had a higher likelihood of all evaluated physical comorbidities and mental health conditions compared to patients without IRD.

To our knowledge, this is the first study to evaluate overall survival among patients with IRD in the US. Globally, only one study has previously estimated overall survival among patients with IRD, using population-based data from South Korea to determine the risk of mortality among patients with retinitis pigmentosa [18]. Data were obtained from the Korean National Health Insurance system, the Rare Intractable Disease registry, and Statistics Korea, allowing for a comprehensive evaluation of mortality rates and causes of death among both patients with retinitis pigmentosa and the general population. The standardized mortality rate (SMR) was 1.56 (95% CI: 1.27–1.90) when considering all patients with retinitis pigmentosa relative to the general population and was highest among males with retinitis pigmentosa aged 40–59 years (SMR: 2.61, 95% CI: 1.60–4.04). The most common causes of death among patients with retinitis pigmentosa were neoplasms, diseases of the circulatory system, and suicide, accounting for 26%, 17%, and 9% of deaths, respectively, and were found to occur at descriptively higher rates among patients with retinitis pigmentosa than the general population. Though not statistically significant due to the small sample size, the risk of mortality associated with most physical comorbidities and mental health conditions trended consistently higher among patients with retinitis pigmentosa versus the general population across age- and sex-based subgroups.

A consistent association between visual impairment and both physical comorbidities and mental health conditions has also been identified in prior research [15,22,23]. In a real-world claims-based study involving nearly 25,000 patients in the US, adults aged 18–64 years with visual impairment were found to have approximately twice the risk of developing neuropsychiatric, musculoskeletal, and cardiometabolic conditions compared to their peers without visual impairment [23]. Further, the Lancet Global Health Commission on Global Eye Health recently proposed that vision impairment may cause or exacerbate physical comorbidities and mental health conditions indirectly through reduced healthcare access, limited physical activity levels, or increased social isolation [24]. Indeed, patients with visual impairment have been shown to experience limitations in mobility and activity, which further hinders their overall health [14]. Research has also highlighted a positive feedback loop between self-reported visual difficulty and vision-related anxiety, whereby self-reported visual difficulty increases vision-related anxiety, which subsequently increases self-reported visual difficulty [25].

Patients with IRD may be at a particularly high-risk of developing physical comorbidities and mental health conditions due to the chronic and untreatable nature of the disease [26,27]. Patients with an IRD-related diagnosis in Taiwan were more likely to experience metabolic comorbidities, such as hypertension, diabetes, and myocardial infarction, compared to patients without IRD [19]. Likewise, the risk of all physical comorbidities evaluated in the current study, including chronic pulmonary disease, diabetes, and peripheral vascular disease, was greater among patients with IRD compared to those without. Notable psychosocial impacts associated with IRD include loneliness and isolation, challenges related to educational attainment and financial success, difficulty living independently, and reduced quality of life compared to the general population [28]. The present study identified a greater risk of anxiety disorders, depression, suicidal attempt or ideation, and trauma and stressor-related disorders among patients with IRD versus without IRD, corroborating the results of previous real-world studies [28]. Given the connection between IRD and both physical comorbidities and mental health conditions, physical and psychosocial impacts should be addressed as part of the complex clinical disease management of this patient population [29,30].

As a result of the early age of IRD onset, this study has implications for the working-age population [5]. Vision loss due to IRD can impact not only the ability to work, but also to participate in activities of daily living [28,31]. Subsequently, a more sedentary lifestyle and decreased quality of life among patients with IRD could further predispose this population to physical comorbidities and mental health conditions and may, in part, explain the lower overall survival among patients with versus without IRD observed in this study.

The high clinical burden of IRD demonstrated in this study highlights the need for novel treatments to maintain or improve vision among affected patients. Voretigene neparvovec-rzyl (Luxturna) is the only gene therapy approved by the US FDA for the treatment of an IRD [8]. Approval as therapy for patients with biallelic RPE65 mutation-associated retinal dystrophy was granted in 2017 after patients demonstrated an improved ability to navigate in low-to-moderate light conditions following voretigene neparvovec-rzyl treatment in a phase 3 randomized, controlled trial [8,9]. Additional treatments are in development and have shown promising initial results, including encouraging improvements in retinal sensitivity, visual function, and functional vision [11]. Botaretigene sparoparvovec treatment, for example, was recently shown to improve retinal sensitivity and functional vision among patients with retinitis pigmentosa GTPase regulator (RPGR)-associated X-linked retinitis pigmentosa in a phase 1/2 trial [32]. There are currently numerous ongoing clinical trials for IRD with the potential to not only prevent vision loss but also avoid secondary comorbidities associated with vision impairment-related lifestyle changes.

### Limitations

This study was associated with several limitations. Analyses of EHR databases are limited by data availability and depend on both correct diagnosis codes and the accuracy of clinical data. Any potential coding errors or missing data may lead to misclassifications. As the study population comprised patients aged <65 years, the results may not be generalizable to older patients with IRD. Due to the observational retrospective nature of the study design, findings may only suggest associations between patients with IRD and overall survival, as well as between IRD status and the risk of physical comorbidities or mental health conditions, and cannot determine causation. As a result, it is not possible to distinguish whether comorbidities preceded or followed IRD diagnosis. Accordingly, the analyses reflect observed prevalence rather than incident comorbidity onset following IRD diagnosis. In addition, a subset of IRDs are syndromic in nature and may be associated with health conditions beyond ocular involvement. As a result, the observed frequency of physical comorbidities and mental health conditions among patients with IRD may, in part, reflect the presence of syndromic forms of IRD, which were not examined separately in this analysis. This analysis balanced for differences in baseline patient characteristics using a matching approach; however, the potential for residual confounding still exists due to unmeasured confounders, particularly when effect sizes are small. The index date among the IRD cohort was defined as the second medical visit with a diagnosis for IRD to ensure IRD was accurately diagnosed and minimize misclassification due to a rule-out diagnosis. Although this may implicate a possible survival bias as well as a cohort that seeks more medical care, the risk of mortality was still found to be higher among patients with IRD versus without IRD in the present analysis. Finally, the index date among the non-IRD cohort was defined by a randomly selected medical visit, which may have led to a selection bias based on the criteria that patients must have sought medical care; however, given the study intake period was approximately 7 years, it can be assumed that members of the general US population would seek any type of medical care within a 7-year period.

## 5. Conclusions

In this real-world study, patients aged <65 years with IRD had a significantly higher risk of all-cause mortality compared to those without IRD. Moreover, the prevalence of common physical comorbidities and mental health conditions was greater in the IRD cohort compared to the non-IRD cohort. These findings suggest that the management of IRD must extend beyond conventional ophthalmic care, emphasizing the importance of addressing the overall clinical presentation and well-being of this patient population.

## Figures and Tables

**Table 1:** Patient demographic characteristics evaluated on the index date.

	Unmatched			Matched		
	IRD Cohort	Non-IRD Cohort	Std. Diff. (%)	IRD Cohort	Non-IRD Cohort	Std. Diff. (%)
	N = 4594	N = 4,205,469		N = 4594	N = 9188	
Age (years), mean ± SD [median]	38.7 ± 18.6 [42.2]	36.1 ± 17.9 [36.9]	14.5	38.7 ± 18.6 [42.2]	38.2 ± 18.5 [40.8]	2.6
Age categories, n (%)						
<18	880 (19.2)	829,848 (19.7)	1.5	880 (19.2)	1759 (19.1)	0.0
18–35	920 (20.0)	1,136,198 (27.0)	16.5	920 (20.0)	1945 (21.2)	2.8
36–49	1139 (24.8)	1,043,604 (24.8)	0.1	1139 (24.8)	2258 (24.6)	0.5
50–64	1655 (36.0)	1,195,819 (28.4)	16.2	1655 (36.0)	3226 (35.1)	1.9
Sex at birth, n (%)						
Female	2476 (53.9)	2,353,407 (56.0)	4.1	2476 (53.9)	5067 (55.1)	2.5
Male	2116 (46.1)	1,849,229 (44.0)	4.2	2116 (46.1)	4114 (44.8)	2.6
Unknown	2 (0.0)	2833 (0.1)	1.0	2 (0.0)	7 (0.1)	1.3
Race, n (%)						
Caucasian	3457 (75.3)	2,999,450 (71.3)	8.9	3457 (75.3)	6850 (74.6)	1.6
African American	535 (11.6)	498,041 (11.8)	0.6	535 (11.6)	1055 (11.5)	0.5
Asian	140 (3.0)	106,776 (2.5)	3.1	140 (3.0)	295 (3.2)	0.9
Other/Unknown	462 (10.1)	601,202 (14.3)	13.0	462 (10.1)	988 (10.8)	2.3
Ethnicity, n (%)						
Not Hispanic	3849 (83.8)	3,282,802 (78.1)	14.6	3849 (83.8)	7655 (83.3)	1.3
Hispanic	323 (7.0)	314,300 (7.5)	1.7	323 (7.0)	650 (7.1)	0.2
Unknown	422 (9.2)	608,367 (14.5)	16.4	422 (9.2)	883 (9.6)	1.5
Region, n (%)						
Midwest	2522 (54.9)	2,005,657 (47.7)	14.4	2522 (54.9)	4986 (54.3)	1.3
Northeast	746 (16.2)	601,033 (14.3)	5.4	746 (16.2)	1530 (16.7)	1.1
South	668 (14.5)	899,186 (21.4)	17.8	668 (14.5)	1344 (14.6)	0.2
West	433 (9.4)	455,485 (10.8)	4.7	433 (9.4)	872 (9.5)	0.2
Other/Unknown	225 (4.9)	244,108 (5.8)	4.0	225 (4.9)	456 (5.0)	0.3
Insurance type, n (%)						
Commercial	2129 (46.3)	1,739,131 (41.4)	10.1	2129 (46.3)	4317 (47.0)	1.3
Dual Insurance	737 (16.0)	253,474 (6.0)	32.0	737 (16.0)	1639 (17.8)	4.8
Medicare	377 (8.2)	60,532 (1.4)	31.6	377 (8.2)	499 (5.4)	11.0
Medicaid	512 (11.1)	317,737 (7.6)	12.3	512 (11.1)	1082 (11.8)	2.0
Uninsured, other payer type, or unknown	839 (18.3)	1,834,595 (43.6)	54.9	839 (18.3)	1651 (18.0)	0.8
Year of index date, n (%)						
2015–2017	1879 (40.9)	2,151,820 (51.2)	20.6	1879 (40.9)	3749 (40.8)	0.2
2018–2019	1632 (35.5)	1,092,653 (26.0)	20.7	1632 (35.5)	3146 (34.2)	2.7
2020–2022	1083 (23.6)	960,996 (22.9)	1.7	1.083 (23.6)	2293 (25.0)	3.2

Abbreviations: IRD: inherited retinal disease; SD: standard deviation; Std. Diff.: standardized difference.

**Table 2:** Patient physical comorbidities and mental health conditions during the 12-month baseline period.

	Unmatched			Matched		
	IRD Cohort	Non-IRD Cohort	Std. Diff. (%)	IRD Cohort	Non-IRD Cohort	Std. Diff. (%)
	N = 4594	N = 4,205,469		N = 4594	N = 9188	
Quan-CCI, ^1^ mean ± SD [median]	0.5 ± 1.1 [0.0]	0.2 ± 0.8 [0.0]	25.4	0.5 ± 1.1 [0.0]	0.4 ± 1.0 [0.0]	10.8
Physical comorbidities, ^2^ n (%)						
Chronic pulmonary disease	502 (10.9)	260,347 (6.2)	16.9	502 (10.9)	819 (8.9)	6.7
Diabetes	431 (9.4)	184,351 (4.4)	19.7	431 (9.4)	650 (7.1)	8.4
Stroke (including TIA)	115 (2.5)	35,362 (0.8)	13.0	115 (2.5)	133 (1.4)	7.6
Cerebrovascular disease	110 (2.4)	38,515 (0.9)	11.6	110 (2.4)	138 (1.5)	6.5
Peripheral vascular disease	102 (2.2)	35,704 (0.8)	11.2	102 (2.2)	158 (1.7)	3.6
Congestive heart failure	100 (2.2)	38,553 (0.9)	10.2	100 (2.2)	177 (1.9)	1.8
Myocardial infarction	62 (1.3)	29,718 (0.7)	6.4	62 (1.3)	117 (1.3)	0.7
Mental health conditions, ^2^ n (%)						
Anxiety disorders	559 (12.2)	300,910 (7.2)	17.0	559 (12.2)	899 (9.8)	7.6
Depression	556 (12.1)	270,568 (6.4)	19.5	556 (12.1)	881 (9.6)	8.1
Suicidal attempt or ideation	156 (3.4)	58,993 (1.4)	13.0	156 (3.4)	228 (2.5)	5.4
Trauma and stressor-related disorders	105 (2.3)	57,434 (1.4)	6.9	105 (2.3)	173 (1.9)	2.8

Abbreviations: CCI: Charlson Comorbidity Index; IRD: inherited retinal disease; SD: standard deviation; Std. Diff.: standardized difference; TIA: transient ischemic stroke. Notes: ^1^. Quan-CCI was evaluated from the beginning of the 12-month baseline period to the index date (inclusive). ^2^. Presence of baseline comorbidities and conditions listed are non-exhaustive and not mutually exclusive.

## Data Availability

The data that support the findings of this study are available from Optum^®^. Restrictions apply to the availability of these data, which were used under license for this study.

## Supplementary Materials

The following supporting information can be downloaded at: https://www.mdpi.com/article/10.3390/vision10010015/s1, Table S1: Diagnosis codes for IRD; Table S2: Diagnosis codes of physical comorbidities and mental health conditions. Refs. [33,34,35,36] are cited in the Supplementary Materials.

## Abbreviations

The following abbreviations are used in this manuscript:

CI	Confidence interval
EHR	Electronic health record
FDA	Food and Drug Administration
HIPAA	Health Insurance Portability and Accountability Act
HR	Hazard ratio
IRD	Inherited retinal diseases
KM	Kaplan–Meier
OR	Odds ratio
PSM	Propensity score matching
Quan-CCI	Quan-Charlson Comorbidity Index
RPGR	Retinitis pigmentosa GTPase regulator
SD	Standard deviation
SMR	Standardized mortality rate
TIA	Transient ischemic attack
US	United States
