# Supplementary material for: Real-World Comparison of Overall Survival Among Patients With and Without Inherited Retinal Diseases
Source: Vision (Basel). 2026 Mar 1;10(1):15. doi: 10.3390/vision10010015 (PMC13030414; doi:10.3390/vision10010015)
Supplement: Supplementary file 1 [file vision-10-00015-s001.zip › vision-4115411-supplementary.pdf]

Table S1. Diagnosis codes for IRD [33].

Description	ICD-9-CM	ICD-10-CM
**I. Photoreceptor disease**		
**A. Isolated**		
**1. Acquired/Progressive**		
a. Retinitis pigmentosa	362.74	H35.52
b. Cone and cone-rod dystrophy	362.75	H35.53
**2. Congenital/stationary**		
a. Leber congenital amaurosis	362.70	H35.50
b. Achromatopsia (congenital stationary cone dysfunction)	368.54	H53.51
c. Blue cone monochromacy	368.54	H53.51
d. Other retinal changes, congenital	743.56	
**B. Syndromic**		
**1. Usher syndrome**		
a. Type I		
b. Type II	362.70-362.75, 362.77	H35.50-H35.53
c. Type III		
**2. Bardet-Biedl syndrome**	759.89	Q87.89
**3. Retinitis pigmentosa with ataxia**	277.87	E88.49
**II. Macular dystrophies**		
**A. Autosomal recessive Stargardt disease**	362.70, 362.75	H35.50, H35.53
**B. Best disease**	362.70	H35.50
**C. Autosomal dominant Stargardt disease**	362.70, 362.75	H35.50, H35.53
**D. Malattia leventinese**	362.70-362.75, 362.77	H35.50-H35.53
**E. North Carolina macular dystrophy**	362.70-362.75, 362.77	H35.50-H35.53
**III. Third branch disorders**		
**A. Choroidopathies**		
**1. Choroideremia**	363.55	H31.21

Abbreviations: ICD-9-CM: International Classification of Diseases, 9th Revision, Clinical Modification; ICD-10-CM: International Classification of Diseases, 10th Revision, Clinical Modification; IRD: inherited retinal disease.

Table S2. Diagnosis codes for physical comorbidities and mental health conditions.

Condition	ICD-9-CM	ICD-10-CM
**Physical comorbidities**		
Chronic pulmonary disease[1]	416.8, 416.9, 490–505, 506.4, 508.1, 508.8	I27.8, I27.9, J40–J47, J60–J67, J68.4, J70.1, J70.3
Diabetes (with and without chronic complications)[1]	250.00–250.3, 250.4–250.9	E10, E11, E12, E13, E14
Peripheral vascular disease[1]	093.0, 437.3, 441, 443.1–443.9, 447.1, 557.1, 557.9, V43.4	I70, 171, I73.1, I73.8, I73.9, I77.1, I79.0, I79.2, K55.1, K55.8, K55.9, Z95.8, Z95.9
Congestive heart failure[1]	398.91, 402.01, 402.11, 402.91, 404.01, 404.03, 404.11, 404.13, 404.91, 404.93, 425.4–425.9, 428	I09.9, I11.0, I13.0, I13.2, I25.5, I42.0, I42.5–I42.9, I43, I50, P29.0
Stroke including transient ischemic attack	V12.54, V17.1, 430-432, 434.00, 434.01, 434.10, 434.11, 434.90, 434.91, 433.00, 433.10, 433.20, 433.30, 433.80, 433.90, 437	I70, 171, I73.1, I73.8, I73.9, I77.1, I79.0, I79.2, K55.1, K55.8, K55.9, Z95.8, Z95.9
Cerebrovascular disease[2]	362.34, 430.x–438.x	G45.x, G46.x, H34.0, I60.x–I69.x
Myocardial infarction[2]	410.x, 412.x	I21.x, I22.x, I25.2
**Mental health conditions**		
Anxiety disorders[3]	293.84, 300.23, 300.00, 300.01, 300.02, 300.09, 300.22, 300.29, 309.21, 312.23	F93.0, F94.0, F40.218, F40.228, F40.230, F40.231, F40.232, F40.233, F40.248, F40.298, F40.10, F41.0, F40.00, F41.1, F06.4, F41.8, F41.9
Depression	296.2, 296.3, 296.5, 300.4, 309, 311	F20.4, F31.3–F31.5, F32, F33, F34.1, F41.2, F43.2
Suicidal attempt or ideation	E950, E951, E952, E953, E954, E955, E956, E957, E958, E959, V62.84	T14.91x, X71.x-X83.x, T36.x-T65.x, T71.x, R45.851
Trauma and stressor-related disorders[3]	313.89, 309.81, 308.3x, 309.0x, 309.24, 309.28, 309.3x, 309.4x, 309.9x, 309.89	F94.1, F94.2, F43.10, F43.0, F43.21, F43.22, F43.23, F43.24, F43.25, F43.20, F43.9, F43.8

Abbreviations: ICD-9-CM: International Classification of Diseases, 9th Revision, Clinical Modification; ICD-10-CM: International Classification of Diseases, 10th Revision, Clinical Modification; IRD: inherited retinal disease.

Notes:
1. Identified using the Elixhauser definition [34].
2. Identified using the Quan-Charlson definition [35].
3. Identified using the DSM-5 definition [36].
